# Protective effects of phenolic acids on mercury-induced DNA damage in precision-cut kidney slices

**DOI:** 10.22038/ijbms.2019.30056.7242

**Published:** 2019-04

**Authors:** Irma Edith Carranza-Torres, Ezequiel Viveros-Valdez, Nancy Elena Guzmán-Delgado, Sara García-Davis, Javier Morán-Martínez, Nadia Denys Betancourt-Martínez, Isaías Balderas-Rentería, Pilar Carranza-Rosales

**Affiliations:** 1 Departamento de Biología Celular y Ultraestructura, Centro de Investigación Biomédica, Facultad de Medicina, Universidad Autónoma de Coahuila. Torreón, Coah. México; 2 Departamento de Biología Celular y Molecular, Centro de Investigación Biomédica del Noreste, Instituto Mexicano del Seguro Social, Monterrey, NL. México; 3 Departamento de Química Analítica, Facultad de Ciencias Biológicas, Universidad Autónoma de Nuevo León, San Nicolás de los Garza, NL. México; 4 División de Investigación, Unidad Médica de Alta Especialidad # 34, Instituto Mexicano del Seguro Social, Monterrey, NL. México; 5 Laboratorio de Ingeniería Genética y Genómica, Facultad de Ciencias Químicas, Universidad Autónoma de Nuevo León, San Nicolás de los Garza, NL. México

**Keywords:** Comet assay, Genotoxicity, Mercuric chloride, Phenolic compounds, Precision-cut tissue slices

## Abstract

**Objective(s):**

Precision-cut tissue slices are considered an organotypic 3D model widely used in biomedical research. The comet assay is an important screening test for early genotoxicity risk assessment that is mainly applied on in vitro models. The aim of the present study was to provide a 3D organ system for determination of genotoxicity using a modified method of the comet assay since the stromal components from the original tissue make this technique complicated.

**Materials and Methods:**

A modified comet assay technique was validated using precision-cut hamster kidney slices to analyze the antigenotoxic effect of the phenolic compounds caffeic acid, chlorogenic acid, and rosmarinic acid in tissue slices incubated with 15 µM HgCl_2_. Cytotoxicity of the phenolic compounds was studied in Vero cells, and by morphologic analysis in tissue slices co-incubated with HgCl_2_ and phenolic compounds.

**Results:**

A modification of the comet assay allows obtaining better and clear comet profiles for analysis. Non-cytotoxic concentrations of phenolic acids protected kidney tissue slices against mercury-induced DNA damage, and at the same time, were not nephrotoxic. The highest protection was provided by 3 µg/ml caffeic acid, although 6 µg/ml rosmarinic and 9 µg/ml chlorogenic acids also exhibited protective effects.

**Conclusion:**

This is the first time that a modification of the comet assay technique is reported as a tool to visualize the comets from kidney tissue slices in a clear and simple way. The phenolic compounds tested in this study provided protection against mercury-induced genotoxic damage in precision-cut kidney slices.

## Introduction

Precision-cut tissue slices represent a three-dimensional (3D) biological model closely resembling the organ from which they are prepared, where all cell types and tissue architecture are preserved, as well as metabolic activity and transport mechanisms (1-3). These characteristics are very treasured in the investigation of different biological activities. Several studies show that precision-cut kidney slices (PCKS) are a useful model for studying drug metabolism, nephrotoxicity, cryopreservation, and fibrosis (4-8) among other applications. 

The comet assay is a quantitative technique through which visual evidence of DNA damage in individual eukaryotic cells can be measured. This method possesses a number of advantages when compared to other genotoxicity tests; one of them being that it´s readily modifiable for adaptation to a variety of experimental requirements. Over time, many improvements and variations of the method have been developed for a number of experimental purposes (9, 10); however, new applications for this method require standardization. A small number of DNA damage studies using the comet assay in cells from precision-cut tissue slices have been reported as tools for genotoxic compounds testing (1, 11-14). In these studies, the comet assay was used to study genotoxicity induced by several xenobiotics or nanomaterials in liver and lung slices (1, 12-14); and, to evaluate the antigenotoxic effects of flavonoids (11). Depending on the kind of tissue used, different modifications were made. For example, human or murine tissue slices were prepared for the assay by mincing the tissue (14), enzymatic digestion was applied to murine lung slices (12), and human and rat liver slices were directly gently squashed with a coverslip (1, 11, 13). These reports demonstrated the usefulness of 3D tissue culture models for genotoxicity studies given that the main advantage of the said models is that the results obtained are comparable to *in vivo* conditions. So far, PCKS studies aimed at determining the degree of protection offered by bioactive compounds against genotoxic damage have not been published. 

On the other hand, large population groups of people are currently exposed to low levels of xenobiotics such as mercury, which has been implicated in the rising prevalence of autism spectrum disorders (15, 16). Occupational or environmental exposure to mercury causes many other health complications (17). Mercuric chloride (HgCl_2_) toxicity is related to oxidative stress (18, 19), it is well known that it is genotoxic at low doses (20), and may be a primary cause of precancerous injuries (21). For these reasons, there is an increasing interest in naturally occurring antioxidants capable of acting as chelating agents and free-radical scavengers against heavy metal poisoning (22). In relation to this, phenolic acids (PAs) are free-radical scavengers universally distributed in the kingdom Plantae; they are essential for plant growth, reproduction, and serve as a defense mechanism against pathogens (23). The antioxidant activity of phenolic compounds and their possible uses in processed foods and treatments against many different diseases have been reported (24, 25). In previous studies, we reported on the anti-radical and antiproliferative activities against cancer cell lines of the phenolic compounds caffeic acid (CA), rosmarinic acid (RA), and chlorogenic acid (CGA) (26, 27). These PAs are present in a variety of species used to make teas, as well as other edible, and medicinal plants, and exhibit diverse biological activities (28-30). In recent years, they have received particular attention due to their role in the prevention of several human diseases. 

In this study, we made an adaptation to the comet assay technique in order to better analyze the protective effects of CA, RA, and CGA on the genotoxic damage induced by HgCl_2_ using the PCKS model. Our results show that the modification made to the comet assay enabled us to analyze comets in tissue slices in an easy and clear manner and that PAs protected kidney tissue slices against mercury-induced DNA damage, and that they were not nephrotoxic. 

## Materials and Methods

***Chemicals***

Mercuric chloride (HgCl_2_), phenolic compounds (CA, RA, and CGA), normal, and low-melting-point agaroses, 1, 1-diphenyl-2-picrylhydrazyl (DPPH), 4-[3-(4-iodophenyl)-2-(4-nitrophenyl)-2H-5-tetrazolium]-1,3-benzene disulfonate (WST-1), and other chemicals were purchased from Sigma-Aldrich Corp. (St. Louis, MO, USA). Cell culture medium and fetal bovine serum (FBS) were obtained from Gibco Invitrogen (Carlsbad, CA, USA).

***1, 1-diphenyl-2-picrylhydrazyl (DPPH) radical-scavenging activity***

Before experiments with tissue slices and comet assay, free radical scavenging activity of the PAs was confirmed. To do this, serial dilutions of PAs dissolved in methanol were mixed with DPPH solution (2 mg/l) in 96-well microplates and incubated for 30 min at room temperature. The change in absorbance at 517 nm was measured. Mean values were obtained from triplicate experiments. The percentage of inhibition was calculated using the following equation: 

% inhibition = [(*A0* − *A1*)/*A0*] × 100, where *A0* is the absorbance of the control and *A1* is the absorbance of the samples (26). 

Radical-scavenging activities were expressed as 90% inhibitory concentrations (IC_90_), and a smaller value indicated more effective radical scavenging. IC_90_ values were calculated from log-dose inhibition curves obtained using a nonlinear regression algorithm (Prism 4.0; GraphPad Software, Inc., La Jolla, CA, USA).

***Cell line and culture***

Vero cells (kidney epithelial cells; American Type Culture Collection: CCL 81) were used to determine the nontoxic doses of the phenolic compounds to be used in PCKS. Cells were grown in M199 medium supplemented with 5% (v/v) FBS, penicillin G (100 units/ml), and streptomycin (100 µg/mL). Cultures were maintained at 37 ^°^C in a humidified 5% CO_2_ atmosphere.

***Cytotoxicity assay***

Vero cells were plated in 96-well flat-bottomed plates at 5000 cells/well and allowed to adhere overnight. Subsequently, cells were treated with different concentrations of the compounds (0–50 μg/ml). After 24 hr, the WST-1 colorimetric assay was used to monitor cell growth (27). Cell growth percentage was used to obtain dose-response curves and determine the 10% inhibitory concentration (IC_10_). Values were expressed as the percentage variation relative to the control. Cellular inhibition was measured as a function of inhibitory concentration, and a higher IC_10_ value indicated lower cytotoxicity.

**Table 1:** Radical scavenging and cytotoxic activities of phenolic acids

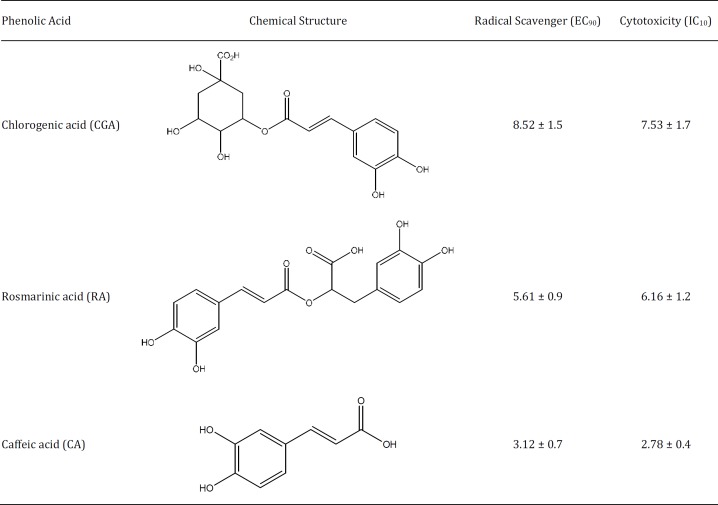

Data show the amount of PAs needed to capture 90 percent of free radicals and inhibit only 10 percent of normal kidney cells (non-cytotoxic doses). CA possesses the best radical-scavenging activity but also is the most cytotoxic. Values (µg/ml) represent the mean and standard deviation of triplicate measurements

**Figure 1:**
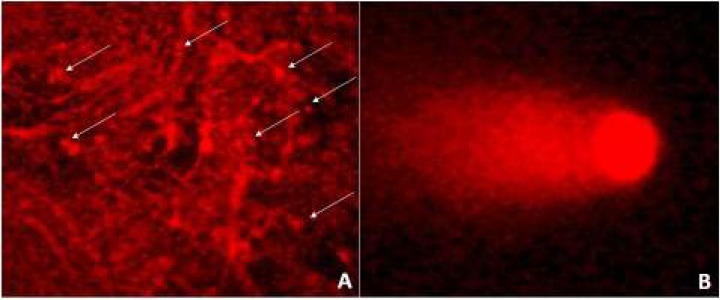
Fluorescent microscope images of tissue slices for the comet assay

PCKS were incubated with 15 µM HgCl2 and processed for comet assay with the conventional technique (a), where a mass of tissue contains many renal cells trapped on the stroma (arrows) which are not appropriated for visual scoring after the squashing method. (b) Cells released from a treated slice after completion of the procedure using the homogenization method with the saccharose buffer. Ethidium bromide stain. Total magnification A (100X), B (400X)

**Figure 2:**
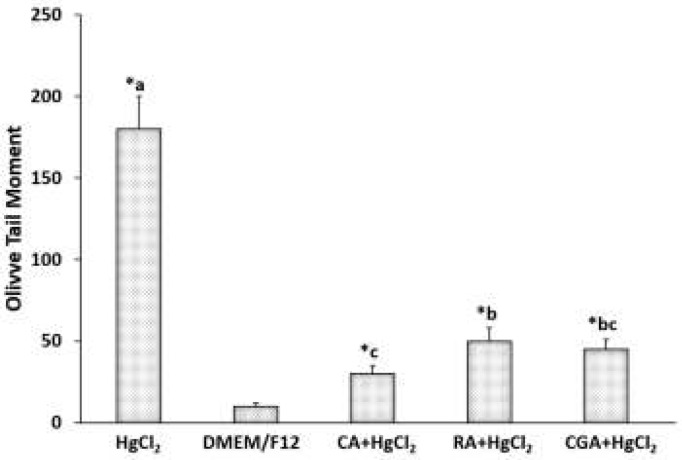
Olive tail moment of precision-cut kidney slices exposed to mercuric chloride and treated with phenolic acids

The olive tail moment was determined from each of the treatments. HgCl_2_ (15 µM) was used as control of damage; negative control was DMEM/F12 medium. Kidney slices were co-incubated separately with 15 µM HgCl_2_ and CA (3 µg/ml), RA (6 µg/ml) and CGA (9 µg/ml). Values represent the mean and standard deviation of 100 cells/ treatment. Normality of the data was evaluated with the Shapiro-Wilks test. The T Student’s Test was applied, and significant differences were analyzed with the Tukey’s test (*P≤*0.01). A significant difference exists between slices treated with HgCl_2 _(a) and those co-incubated with the PAs (b, c, d). CA exhibited the biggest difference (c)

**Figure 3:**
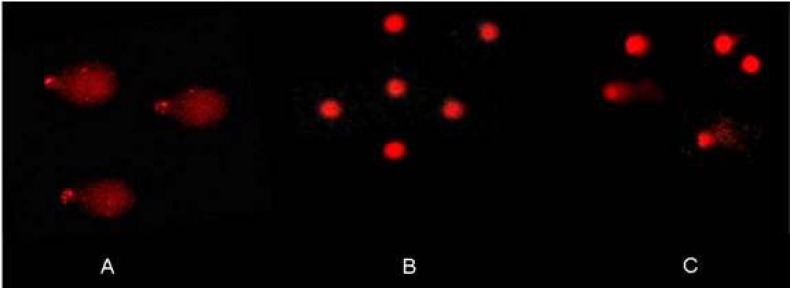
Comet assay from kidney slices incubated with mercuric chloride and phenolic acids

Representative cell images from PCKS incubated with 15 µM HgCl_2_ showed the typical form of long-tail comets indicating DNA strand breaks in the cells (a). Untreated slices (b) showed round nuclei, with intact DNA, while in the kidney slices incubated with 3 µg/mL CA+15 µM HgCl_2_ the damage was attenuated (c). Ethidium bromide stain. Total magnification (200X)

**Figure 4:**
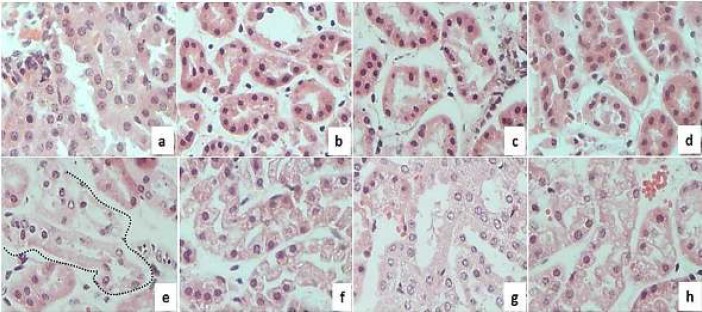
Histopathologic analysis of precision-cut kidney slices treated with mercuric chloride and phenolic acids

PCKS were incubated in the presence of (a) cell culture medium, (b) 9 µg/ml CGA, (c) 3 µg/ml CA, (d) 6 µg/ml RA, (e) 15 µM HgCl_2_, (f) CGA+HgCl_2_, (g) CA+HgCl_2_, and (h) RA+HgCl_2_. Untreated slices showed morphologic characteristics of normal tissue, as well as preserved tubular structure (a). Slices incubated in the presence of phenolic acids alone show no signs of damage or morphological alterations (b, c, d). In contrast, mercury induced interstitial edema, tubular necrosis and the presence of apoptotic nuclei (e), dotted lines. In slices co-incubated with HgCl_2_ and compounds, the damage induced by mercury was ameliorated; this was evident because of the conserved histologic structure, showing only mild interstitial edema. With CGA and RA hydropic degeneration was observed, whereas, with CA, less degenerative changes were observed (f, g, h). H&E stain. Total magnification 400X

***Preparation of precision-cut kidney slices***

PCKS were prepared from two month old male Syrian golden hamsters (*Mesocricetus auratus*). Hamsters were killed with an overdose of sodium pentobarbital (6 mg/100 g) and treated following institutional and international guidelines for the humanitarian care of animals used in experimental work. Once the hamsters were unconscious, the kidneys were quickly removed and placed into ice-cold Krebs–Henseleit buffer (KB buffer). The external membranes covering the kidneys were separated with a scalpel, and the organs were cored to yield cylinders of 5 mm diameter. The cores were then sliced in oxygenated KB buffer (4 ^°^C, 95:5 O_2_:CO_2_) into ~150-µm-thick slices, using a Brendel–Vitron tissue slicer (Vitron Inc., Tucson, AZ, USA). After preparation, slices were kept in ice-cold KB solution until incubation. The whole procedure from organ excision to incubation typically took 2 hr. The maintenance and care of the experimental animals complied with Instituto Mexicano del Seguro Social and Ley General de Salud (México) regulations and international guidelines for the humane use of laboratory animals.

***Incubation of PCKS***

PCKS were cultured in 24-well culture plates (Corning Life Sciences, Acton, MA, USA) in Dulbecco’s modified Eagle’s medium/F12 serum-free medium (1.3 ml/well) supplemented with glucose (25 mM) and gentamicin (50 μg/ml) at 37 ^°^C in a 95% air/5% CO_2_ atmosphere. The slices were incubated for 30 min in culture medium (untreated control) with individual PAs (9 µg/mL CGA, 3 µg/ml CA, 6 µg/ml RA), or 15 µM HgCl_2_, and co-incubated with HgCl_2_ plus each PA (CGA+HgCl_2_, CA+HgCl_2_, and RA+HgCl_2_) at the same concentration mentioned above. During incubation, plates were stirred at 30 rpm to facilitate gas exchange and substrate diffusion in the slices and ensure continuous floating of the slices in the medium. All experiments were performed with slices from at least three different experiments, and duplicate slices were used for each experimental condition.

***Preparation of slides for the comet assay***

We made a modification to the comet technique in order to obtain separated nuclei from PCKS- cells as well as cells maintaining their structural integrity. To do this a mechanical homogenization was performed according to the method described by Hymer and Kuff (31), however, a filtration step was not applied to the homogenates and the use of detergents was avoided. The method used in the present work is described below. Fully frosted microscope slides were pre-coated with a layer of 0.75% normal agarose. After incubation with the compounds, the PCKS were homogenized using a Potter–Elvehjem tissue grinder (Fisher Scientific, Hampton, New Hampshire, USA) in saccharose buffer (0.25 M saccharose and 1 mM MgCl_2_) (31). Once partially disintegrated, the material was centrifuged at 5000 rpm for 10 min, and pellets containing nuclei and cells were resuspended in 90 µL of low-melting-point agarose (0.75%) and placed on the normal agarose layer. Next, coverslips were placed on top of the samples, and each cellular pellet was squashed to release cells. The liberated nuclei and cells spread over the microscope slide but remained embedded in the agarose. Slides were kept at 4 ^°^C for up to 5 min to allow the agarose to solidify, after which the coverslips were removed. Finally, each preparation was covered with a layer of low-melting-point agarose. 

***Comet assay***

The subsequent steps of the comet assay were performed as described by Singh *et al.* (32). Previously prepared slides were incubated in cold lysis solution [2.5 M NaOH, 0.1 M ethylenediaminetetraacetic acid (EDTA), and 0.01 M Tris with 1% Triton X-100, pH 10] at 4 °C for at least 1 hr. Then, slides were placed in a horizontal electrophoresis box, and the DNA was allowed to unwind for 20 min in freshly prepared alkaline electrophoresis buffer (300 mM NaOH and 1 mM EDTA, pH 13). Electrophoresis was conducted at 4 ^°^C for 20 min at 25 V and 300 mA. Slides were then neutralized with Tris buffer (0.4 M, pH 7.5) and stained with ethidium bromide. Slides were observed under a fluorescence microscope with a charge-coupled device camera and analyzed using an image analysis system (CASP). For each treatment, 100 single cells were analyzed; three separate experiments were conducted for each series. DNA damage was expressed as the olive tail moment, which is defined as the product of the tail length and fraction of total DNA in the tail. The tail moment incorporates a measure of both the smallest detectable size of migrating DNA (reflected in the comet tail length) and the number of relaxed/broken pieces (represented by the intensity of DNA in the tail). The following equation was used:

Olive tail moment=(tail.mean−head.mean) × tail%DNA/100.

It is generally accepted that higher olive moment values indicate more damage to DNA.

***Morphologic analysis***

To study the effects of PAs on renal tissue, PCKS were incubated for 12 hr in culture medium with PAs alone (9 µg/ml CGA, 3 µg/ml CA, and 6 µg/ml RA) or 15 µM HgCl_2_, and co-incubated with mercury and PAs (9 µg/mL CGA+15 µM HgCl_2_, 3 µg/mL CA+15 µM HgCl_2_, and 6 µg/mL RA+15 µM HgCl_2_). After incubation, slices were washed with phosphate-buffered saline solution at 37 ^°^C and fixed with 10% neutral formalin for 24 hr at room temperature. Slices were embedded in paraffin using an automated tissue processor. Subsequently, 5-µm thick sections were prepared from paraffin blocks containing the samples using a microtome. These were then stained with hematoxylin and eosin and studied under a conventional light microscope. Histopathologic criteria were used to establish the morphologic characteristics of normal tissue and were compared with those of slices subjected to different treatments. 

***Statistical analysis***

Statistical significance was determined using one-way analysis of variance followed by the Tukey’s test, comparing all groups with the control group. The results were considered statistically significant at *P*<0.01. Data were expressed as means ± standard deviation.

## Results

***Antioxidant and cytotoxic activities***

Caffeic, rosmarinic, and chlorogenic acids are phenolic compounds that can be found in some herbs and spices and were evaluated for their anti-radical activity by the DPPH method; the highest anti-radical activity was exhibited by CA (IC_90_=3.12 μg/ml), followed by RA and CGA (IC_90_= 5.61 and 8.52 μg/ml, respectively). Regarding cytotoxicity in Vero cells (normal kidney cell line), the nontoxic doses (IC_10_) of CA, RA, and CGA were 3, 6.16, and 7.53 μg/ml, respectively. CA exhibited the highest cytotoxic and anti-radical effects, revealing a direct relationship between anti-radical and cytotoxic properties (Table 1).

***Comet assay in PCKS***

When prepared the tissue slices were for the comet assay, the resulting images showed large masses of stromal tissue and few isolated cells (Figure 1A); due to this, we decided to establish a simpler and faster method to measure the DNA damage in PKCS-cells. In order to accomplish this goal, we mechanically disintegrated the tissue slices by gently homogenizing them in a tissue grinder and using saccharose buffer to prevent the released cells from lysing. Both single nuclei and cells were obtained by this method, we were able to visualize that the said nuclei retained their integrity and would be useful for the purpose of our studies (Figure 1B). 

***Protective effect of phenolic acids*** ***on HgCl***_2_***-induced DNA damage***

In this study, we used PCKS and the comet assay to analyze the protective effect of PAs against the genotoxic damage induced by sub-cytotoxic doses of HgCl_2_. PAs efficiently decreased HgCl_2_-induced damage; and, no statistically significant differences were found relative to the untreated control (Figure 2).

With regard to the morphology of the comets formed under different incubation conditions, CA induced shapes with less DNA damage compared with untreated control slices, PCKS treated with 15 μM HgCl_2_, and slices co-incubated with 3 μg/ml CA+15 μM HgCl_2_ (Figure 3). These results suggest that CA has a greater protective effect against mercury-induced damage in renal tissue.

***Morphological analysis***

To establish whether the phenolic compounds had toxic effects on renal tissue, PCKS were incubated for 12 hr in the presence of the treatments as described in the Materials and Methods section. The results show that compared to the control slices, the phenolic compounds did not induce alterations in the histological structure of the kidney (Figure 4D). In contrast, HgCl_2 _caused interstitial edema, proximal tubular necrosis, and apoptosis (Figure 4E). These results indicate that the three studied phenolic compounds are not nephrotoxic. Furthermore, in PCKS co-incubated with the phenolic compounds+HgCl_2 _(Figure 4F-H), morphological damage was less evident, although slight edema was observed in the interstices of the kidney. CGA (9 μg/ml) and RA (6 μg/ml) induced hydropic regeneration, which is associated with an adaptive response. In the same way as results obtained in the comet assay, CA induced minor morphologic changes and has the most effective protection at the tissue level.

## Discussion

The aim of the present study was to render a simple and reproducible 3D *ex vivo* PCKS model for the determination of mercury-induced genotoxic damage using the comet assay, as well as to analyze the protective effect of PAs in the same model. 

Comet assays directly applied to a complex three-dimensional system without preparation of single cells represent a challenge mainly due to the fact that cells are lying on top of one another, and to stromal components present in the original tissue. In order to adequately visualize the comets obtained from PCKS incubated with the different treatments, it was necessary to disaggregate cells from each tissue slice cells under isotonic conditions in a glass homogenizer in the presence of saccharose buffer to avoid cell lysis (31), this process resulted in purified intact nuclei and cells from renal tissue. Thus, for the first time, we report a modification of the original technique that allowed us to visualize the comets from PCKS in a clear, simple, and reproducible manner for the comet assay. This variation made by our laboratory permits avoiding problems with overlapping comets or false positives made by squashing a great number of cells in tissue slices between the coverslip and glass slide.

On the other hand, since its development by Ostling and Johanson (33), the comet assay is widely used to test the genotoxicity of chemical compounds both *in vitro* and *in vivo*. One advantage of the technique is that cells from various tissues of a wide variety of organisms can be studied (10). However, questions have arisen about the results obtained from cell cultures; in many cases, cells in culture cannot adequately metabolize some toxins because they lack important components of metabolic pathways such as cytochrome P450 (34, 35). This problem is addressed by alternative 3D systems, in which all cell types reside in their original tissue–matrix configuration and metabolic activity is preserved. Consequently, research laboratories have begun to use precision-cut slices to study the genotoxic effects of xenobiotics (12-14) and the protective effects of natural products (11), among other applications. This experimental approach has important advantages over *in vitro* and *in vivo* models. This model closely resembles the *in vivo* tissue function, which is highly desirable for the evaluation of new or existing compounds. In addition, precision-cut tissue slices offer the advantage of greater reproducibility in the experiments carried out with the same organ, it also allows to work simultaneously with different organs from the same animal, being able to obtain a large number of slices that can be cultivated, decreasing the number of experimental animals (36, 37). Few reports describe the use of the comet assay to measure DNA damage in *ex vivo* models such as murine and human liver and lung slices; however, reports show that this assay is a useful tool to study genotoxicity induced by xenobiotics such as tert-Butyl hydroperoxide, benzo (α) pyrene, formalin, 2-Nitrofluorene, and nanomaterials (1, 12-14); and to evaluate antigenotoxic effects of flavonoids (11).

In relation to the protective effect on the radical scavenging activity, the three PAs share the presence of the catechol group (3 ‘, 4’-dihydroxy group) in their structure (Table 1), and it is well known that at low concentrations, some compounds that have catechol groups provide protection to primary cultures of mice cortical neurons against methylmercury induced damage (38). Our results suggest a positive relationship between the radical scavenging effects and cytotoxicity (Table 1). In fact, it has been reported that the antioxidant or pro-oxidant activity of the phenolics depends on their concentration (39). Also, the cytotoxicity of some polyhydroxy benzenes studied by QSAR models indicates parallelism between polyphenol cytotoxicity and the rates of their single-electron oxidation, and points to the leading role of formation of the reactive oxygen their cytotoxicity (40). The catechol group has also the ability to donate electrons, which is related to its antioxidant capacity *in vitro* and *in vivo* (41), as well as for the formation of metal complexes (42). Transitional metals as Hg have a major role in the generation of oxygen free radicals in living organisms, in fact, the toxicity of HgCl_2_ is attributed to the high affinity of Hg (II) to the thiol groups, which depletes cellular proteins such as GSH (43), and therefore increases the production of free radicals that can lead to lipid peroxidation, protein modification, and DNA damage. Compounds acting as chelators like the PAs used in this work, can inactivate metal ions and potentially inhibit the harmful processes related to mercury poisoning (44), which in turn can explain the results observed in the present study on the protective effect by scavenging free radicals. Phenolic compounds may inhibit and/or attenuate the damage generated by oxidative stress via several mechanisms, including the antioxidant and free-radical-scavenging activities and their ability to inhibit, activate, or protect specific enzymes in the body (45). In a similar way, to prevent mercury-induced damage, natural products like plant extracts, essential oils, and edible species have been studied (46, 47); for example, Abarikwu *et al.* (46) demonstrated that oil extracted from *Moringa oleifera* diminishes the negative effects of mercury-induced oxidative stress and decreases malondialdehyde levels and superoxide dismutase and catalase activities in rats. 

Mercury-induced oxidative and renal stress response is studied using different procedures (48) and, although CA, RA, and CGA have been evaluated by several authors using different test systems (49), in the present work we report for the first time the use of an *ex vivo* 3D kidney model, where we find results similar to those found in murine models. For example, researchers (50), observed that pretreatment with methanolic extracts from leaves of *Capparis spinosa*, which is rich in phenolic compounds, restores the healthy histology of renal tissue relative to the damage evident in Albino rats intoxicated with cisplatin. Our results are in accordance with reports that have demonstrated the protective effects of CA against the damage induced by paracetamol, ultraviolet light, hydrogen peroxide, lambda-cyhalothrin, ochratoxin A, and pilocarpine (51-56). It has also been reported that low doses of CA protect against endothelial damage caused by oxidative stress (57). Reportedly, CA protects against damage associated with ochratoxin A, tumor promoter 12-O-tetradecanoylphorbol-13-acetate (TPA), carbon tetrachloride, paraquat, hypochlorite, and hydrogen peroxide (55, 58-62). Similarly, RA protects against the damage caused by pilocarpine, aflatoxin B1, ochratoxin A, hydrogen peroxide, and doxorubicin (56, 63-65). All the reported findings were obtained analyzing murine models and/or 2D cell culture. The genoprotective effects of phenolic acid against HgCl_2_-induced damage seem to be related to their anti-radical activity. This was also proposed by Rao and Sharma (66), who found that vitamin E protects against reproductive damage caused by HgCl_2_ in mice. Rao *et al.* (67) also found that vitamin C protects against genotoxic damage induced by HgCl_2_ in the peripheral blood, which they attributed to the antioxidant activity of the vitamin. 

## Conclusion

We have shown that application of the modified comet assay to 3D *ex vivo* models such as PCKS is a useful and complementary tool for determination of genotoxic damage induced by xenobiotic agents, and to evaluate antigenotoxic compounds. This study also shows that CA, RA, and CGA protect PCKS against mercury-induced genotoxic damage and that these compounds are not nephrotoxic. CA showed the best anti-radical activity, and also protected at the tissue level. 
